# Morphological, Physiological, and Taxonomic Characterization of Actinobacterial Isolates Living as Endophytes of Cacao Pods and Cacao Seeds

**DOI:** 10.1264/jsme2.ME15146

**Published:** 2016-03-05

**Authors:** Romaric Armel Mouafo Tchinda, Thaddée Boudjeko, Anne-Marie Simao-Beaunoir, Sylvain Lerat, Éric Tsala, Ernest Monga, Carole Beaulieu

**Affiliations:** 1 Laboratory of Phytoprotection and Valorization of Plants Resources, Biotechnology Centre—NkolbissonP.O. box 3851, Messa, YaoundéCameroon; 2 Department of Biochemistry, Faculty of Science, University of Yaoundé IP.O. box 812 YaoundéCameroon; 3 Centre SÈVE, Département de biologie, Université de SherbrookeSherbrooke, Québec J1K 2R1Canada; 4 Département de Mathématiques, Université de SherbrookeSherbrooke, Québec J1K 2R1Canada

**Keywords:** *Actinomadura*, biocontrol, cacao pod, endophytic actinobacteria, *Streptomyces*

## Abstract

Vascular plants are commonly colonized by endophytic actinobacteria. However, very little is known about the relationship between these microorganisms and cacao fruits. In order to determine the physiological and taxonomic relationships between the members of this community, actinobacteria were isolated from cacao fruits and seeds. Among the 49 isolates recovered, 11 morphologically distinct isolates were selected for further characterization. Sequencing of the 16S rRNA gene allowed the partition of the selected isolates into three phylogenetic clades. Most of the selected endophytic isolates belonged to the *Streptomyces violaceusniger* clade. Physiological characterization was carried out and a similarity index was used to cluster the isolates. However, clustering based on physiological properties did not match phylogenetic lineages. Isolates were also characterized for traits commonly associated with plant growth-promoting bacteria, including antibiosis and auxin biosynthesis. All isolates exhibited resistance to geldanamycin, whereas only two isolates were shown to produce this antibiotic. Endophytes were inoculated on radish seedlings and most isolates were found to possess plant growth-promoting abilities. These endophytic actinobacteria inhibited the growth of various plant pathogenic fungi and/or bacteria. The present study showed that *S. violaceusniger* clade members represent a significant part of the actinobacterial community living as endophytes in cacao fruits and seeds. While several members of this clade are known to be geldanamycin producers and efficient biocontrol agents of plant diseases, we herein established the endophytic lifestyle of some of these microorganisms, demonstrating their potential as plant health agents.

Plants are externally and internally colonized by a multitude of microorganisms that affect their development. Zilber- Rosenberg and Rosenberg (67) suggested that an individual plant, in association with its epiphytic and endophytic colonizers, represents a holobiont, the combined genomes of which constitute the unit that collectively responds to evolutionary forces. Therefore, it is considered important to gain information on the microbial communities living in close relationships with plants. Endophytic microorganisms are defined as microbial species that invade internal plant tissues without causing disease (11, 23). As endophytes, various bacterial species reside in leaves, stems, and roots as well as in fruits and seeds (4, 16, 53, 62). Bacteria that invade seeds have been attracting attention for their ability to be transmitted to the next generation (11). Some bacterial genera, particularly *Bacillus*, *Pseudomonas*, and *Streptomyces*, have frequently been reported to form endophytic associations with plants (17, 36, 53). Although plant-endophytic microbe interactions remain asymptomatic, accumulating evidence suggests that endophytic organisms contribute to plant health. It has been effectively demonstrated that bacterial residents in plant tissues protect their hosts against plant pathogens and/or promote plant growth. These beneficial effects may be attributed to properties such as the facilitation of plant nutrient uptake, biosynthesis of plant hormones, lowering of the amount of plant-synthesized ethylene, and antagonism against phytopathogens through various mechanisms including antibiosis, niche competition, the production of lytic enzymes, and induction of systemic disease resistance (12, 25, 30, 49).

Cacao (*Theobroma cacao*) is an important crop in several African countries. Four of these countries (Ivory Coast, Ghana, Nigeria, and Cameroon) contribute approximately 70% of the world’s cocoa production (15). Cocoa production in Cameroon generates incomes of more than 152 M€ per year to more than 600,000 producers. In Cameroon, cocoa production is challenged by several plant diseases, particularly black pod caused by *Phytophthora* species. To the best of our knowledge, the endophytic bacterial community of cacao plants has never been investigated in Cameroon. However, the endophytic colonization of cacao seedlings and cacao plants has been reported in other countries for several fungal species (14, 39) as well as bacteria of the genus *Bacillus* (19). Endophytic fungi from *Theobroma* have been shown to confer disease protection against two causal agents of black pod rot disease, *Phytophthora megakarya* and *P. palmivora* (24, 60), while endophytic *Bacillus* strains are known to promote the growth of cacao seedlings (19, 32). Although actinobacteria are common inhabitants of the cacao rhizosphere (4) and pod surfaces (37), the endophytic actinobacterial community of cacao has not yet been examined in detail. However, since half of the known antibiotics from microbial sources originate from actinobacteria (5), this bacterial taxon is worth exploring for the identification of biocontrol agents and plant growth-promoting microorganisms (17, 18). Endophytic actinobacteria, chiefly *Streptomyces* species, have been isolated from other cultivated plants such as wheat (13), rice (62), potato (53), and medicinal plants (34, 48). In some cases, these endophytic actinobacteria were found to protect plants against diseases (55) and promote plant growth (13, 22).

The aim of the present study was to characterize the culturable endophytic actinobacterial population from Cameroon cacao pods and cacao seeds for their taxonomic affiliation and physiological properties, including traits associated with plant growth-promoting ability.

## Materials and Methods

### Isolation of endophytic actinobacteria

Mature cacao pods from ♀T79/467 × ♂SNK13 hybrids were collected from the experimental station of the Cacao Development Corporation (SODECAO) at Mengang (Cameroon) and used as source material for the isolation of endophytic actinobacteria. Three cacao pods were washed thoroughly with tap water and, after removal of the exocarp, seeds were collected and the internal mesocarp was cut into pieces of approximately 125 mm^3^. Pod tissues and seeds were then washed with tap water to remove mucilage. The samples were immersed in 1/140 diluted phenol solution (v/v) for 10 min and rinsed twice with sterile distilled water for 15 min. Surface-sterilized samples were then rinsed twice for 5 min in sterile phosphate buffer (0.1 M, pH 7.0). Pod tissues or seeds were placed aseptically into test tubes containing 0.1 M potassium phosphate buffer (pH 7.0) and incubated at 55ºC for 30 min. A sample (100 μL) of this solution was spread onto ISP-2 plates (56) as a control to verify the sterility of phosphate buffer.

Treated tissues or seeds were aseptically crushed in phosphate buffer and 100 μL of solution was plated onto water agar (1.5% [w/v]) supplemented with nystatin 50 mg L^−1^, cycloheximide 50 mg L^−1^, polymixim 5 mg L^−1^, and penicillin 10 IU mL^−1^. Plates were incubated at 30ºC for up to 2 weeks. Actinobacterial colonies were picked and purified by serial passages on ISP-2 medium supplemented with the same antibiotics and subsequently on ISP-2 medium without antibiotics.

### Cultural and morphological characterization of endophytic actinobacteria

Unless otherwise specified, actinobacteria were grown at 30ºC. The cultural characteristics of the isolates were determined by growing them on ISP-3 and ISP-7 media (56) and MS medium (26). Melanin pigment production was determined on ISP-7 medium. The morphology of spore-bearing hyphae was determined on an ISP-4 culture using the cover-slip method (65). According to their morphological characteristics, the isolates were divided in 11 morphotypes (Table 1) and one representative isolate of each group was characterized in more detail.

### Physiological characterization of endophytic actinobacteria

Actinobacterial isolates were examined for the ability to degrade various substrates, to utilize different carbon or nitrogen sources, and to grow in the presence of inhibitors or under a range of temperatures and pH. The degradation of arbutin, aesculin, l-tyrosine, hypoxanthine, and casein was assessed as described by Williams *et al.* (66). The degradation of starch and gelatin was determined on modified Bennett’s agar (MBA, 28) supplemented with 1% starch or 0.4% gelatin, respectively (65). After a 7-d incubation, plates were flooded with an iodine (starch) or MgCl_2_ (gelatin) solution and the presence of clearing zones around colonies was scored as a positive reaction. The degradation of Tween 80 (1%) (57) and cellulose (1%) (61) was tested. Chitinolytic, chitosanolytic, and pectinolytic activities were detected by the appearance of clear zones around colonies grown on ISP-9 medium supplemented with 1% chitin, chitosan, or pectin, respectively (3).

Carbon sources were added at a final concentration of 1% (w/v) in ISP-9 medium (56), except for organic acids, which were used at a concentration of 0.1% (w/v). The assimilation of nitrogen sources was determined according to Williams *et al.* (66). Growth was scored after 14 d by comparing test plates with negative and positive controls.

Tolerance to temperature and pH and resistance to chemical inhibitors was tested on MBA (66). Growth at pH 4.5, pH 7.0, or pH 9.0 was determined at 30ºC, while growth at 4, 25, 37, and 45ºC was determined at pH 7.0. Plates were incubated for up to 6 weeks. In order to determine the level of resistance to potential inhibitors, MBA was supplemented with one of the following chemicals: phenol (0.1% [w/v]), crystal violet (0.0001% [w/v]), thallium acetate (0.01% [w/v]), potassium tellurite (0.01% [w/v]), cadmium chloride (0.01% [w/v]), cephaloridine (100 μg mL^−1^), lincomycin (100 μg mL^−1^), streptomycin (50 μg mL^−1^), kanamycin (50 μg mL^−1^), rifampicin (1 μg mL^−1^), vancomycin (50 μg mL^−1^), geldanamycin (50 μg mL^−1^), penicillin (10 IU mL^−1^), oleandomycin (1 μg mL^−1^), or NaCl (4, 8, 10, and 13% [w/v]). In these tests, growth was scored after 7 and 14 d. Physiological profile comparisons within isolates were performed by calculating the similarity index (66). A dendrogram establishing the physiological relationships between isolates was established using the software R (50).

### Taxonomic relationships between endophytic actinobacterial isolates

Taxonomic relationships between isolates were determined by comparing their 16S rRNA gene sequences. Genomic DNA was isolated using a method developed for Gram-positive bacteria (47). The 16S rRNA gene was amplified using the universal primers BSF8/20 and BSR1541/20 (42). PCR conditions were: 5 min at 95ºC, followed by 35 cycles at 95ºC for 1 min, at 60ºC for 1 min, and at 72ºC for 2 min. PCR products were sequenced at the Genome Quebec Innovation Centre (Montreal, Canada). Regarding the 16S rRNA gene sequence of each isolate, the BLAST (Basic Local Alignment Search Tool) program (http://www.ncbi.nlm.gov/BLAST) (2) and the Sequence Match of Ribosomal Database Project II release 11 (http://rdp.cme.msu.edu/seqmatch/seqmatch_intro.jsp) (10) were used to determine the nearest phylogenetic neighbor sequences. The tree builder of RDP was used to generate the phylogenetic tree using the weighted neighbor-joining tree building algorithm (8). A bootstrap analysis was performed using 100 resamplings and *Nocardia africana* (AF277198) as the outgroup.

### Antagonistic activity of endophytic actinobacteria

The antagonistic activity of the actinobacteria was evaluated against plant pathogens as well as against a representative of each endophytic isolate. Antagonistic assays against oomycetes and fungi (*P. megakaria*, *P. erythroseptica*, *Fusarium oxysporum*, and *Botrytis cinerea*) were carried out as previously described (3). Antagonistic assays were also performed against a Gram-negative bacterial pathogen (*Agrobacterium tumefaciens*, 59) and against actinobacteria (including *Streptomyces scabiei*, 1).

Geldanamycin was extracted from supernatants of 10-d-old YME cultures (63) and the antibiotic was quantified by high-performance liquid chromatography using a previously developed method (33).

### Examination of traits commonly associated with plant growth-promoting rhizobacteria

The endophytic actinobacterial isolates were screened for their ability to solubilize phosphate using bromophenol blue as an indicator (27). Siderophore production was verified on blue agar CAS medium containing chrome azurol S (CAS) and hexadecyltrimethylammonium bromide (HDTMA) as indicators (52). ACC deaminase activity was determined by monitoring the amount of α-ketobutyrate generated by the hydrolysis of 1-aminocyclopropane-1-carboxylate (ACC, 46). The ability to synthesize indole-3-acetic acid (IAA) was assessed in minimal medium supplemented with l-tryptophan (31). Root growth promotion by the various isolates was tested on radish seedlings cultivated in growth pouches as described previously (31).

### Accession numbers

Partial sequences of the 16S rRNA gene obtained for the 11 isolates of interest were deposited in GenBank with the accession numbers KR856289 to KR856299.

## Results

### Cultural properties of actinobacterial isolates

Forty-nine actinobacterial isolates were obtained, 12 of which were isolated from cacao pods and 37 from cacao seeds. Based on their morphological characteristics, the bacterial isolates obtained were divided into 11 groups (Table 1). Twenty-nine isolates from pods were assigned to the group Rec3, which is characterized by spiral spore chains and an orange basal mycelium on ISP-2 medium. The other eight pod isolates were assigned to the group Rec1 (rectiflexible chains of spores borne on a yellow aerial mycelium). The nine additional groups that were defined as Ref8, Ref12, Ref16a, Ref16b, Ref16c, Ref17, Ref20, Ref22, and RefXX included one to four seed-associated isolates. Groups Ref8, Ref17, and Ref20 included isolates that produce spores, whereas the other seed isolates (Ref12, Ref16a, Ref16b, Ref16c, Ref22, and RefXX) did not produce spores *in vitro* (Table 1).

### Physiological characterization of actinobacterial isolates

**Fig. 1:**
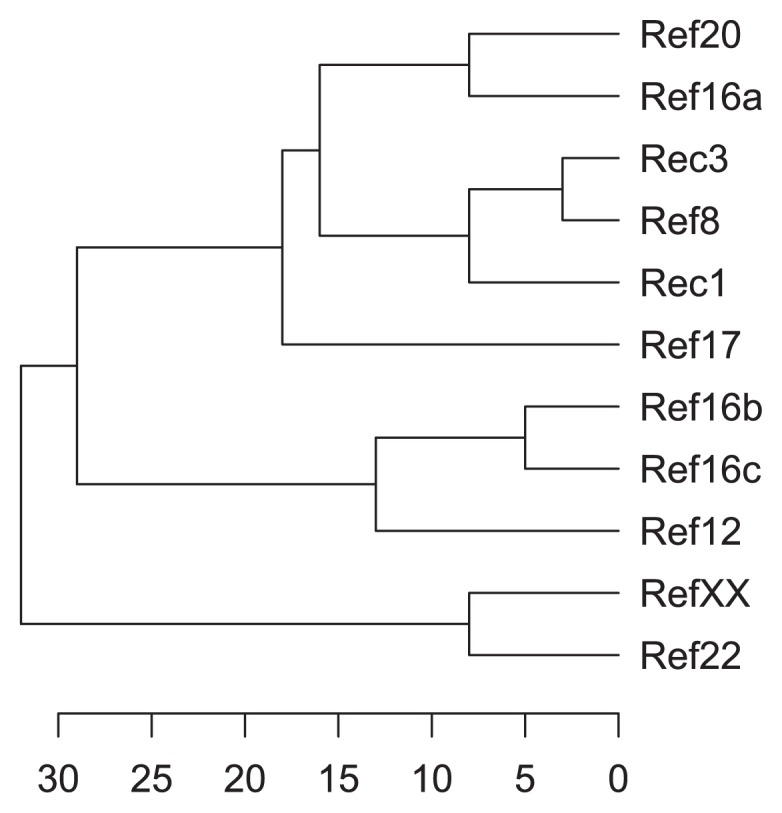
Dendrogram representing physiological profile relatedness among endophytic actinobacteria isolated from cacao pods and cacao seeds. The scale represents the level of dissimilarity (%).

The 11 isolates exhibiting different morphological properties were submitted to a series of 46 physiological tests. Only 17 of these tests allowed discrimination within isolates, the results of which are presented in Table 2 (see also Table S1 for tests that generated identical responses in all isolates). All strains were resistant to the antibiotic geldanamycin (Table S1). The isolates exhibited between 68% and 97% similarity based on their physiological profiles (Table S2). Although their spores were borne in rectiflexible and spiral chains, respectively, pod isolates Rec1 and Rec3 showed very similar physiological profiles (97% similarity, Table S2), discernable only by their abilities to grow at 45ºC (Table 2). The Ref8 seed isolate that exhibited spiral chains of spores was also closely related to the pod isolates Rec1 and Rec3 (92% and 97% similarities, respectively; Table S2). According to their physiological properties, the endophytic actinobacteria were subdivided into three clades (Fig. 1). The first clade included all sporulating bacteria isolated from pods or seeds as well as the non-sporulating Ref16a isolate. All other non-sporulating seed isolates were included in the second (Ref12, Ref16b, and Ref16c) or third clades (Ref22 and RefXX).

### Taxonomical relationship between endophytic actinobacterial isolates

**Fig. 2:**
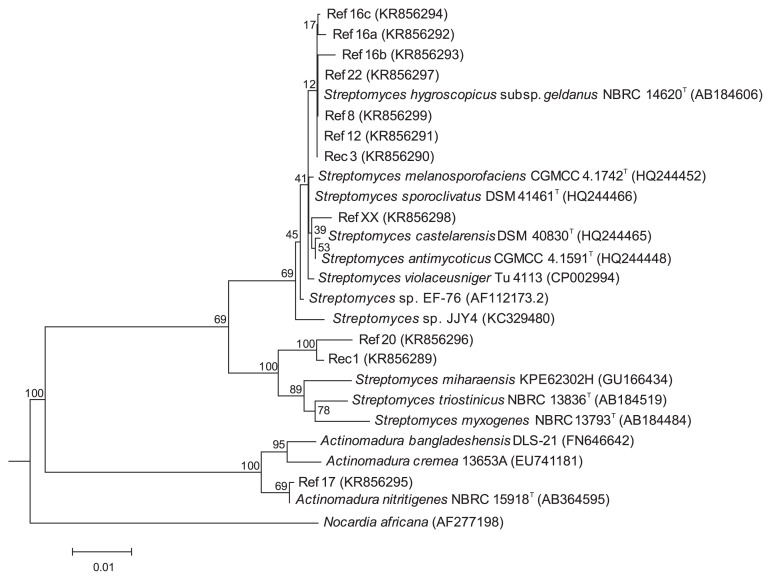
Phylogenetic tree based on 16S RNA gene sequences of endophytic cacao isolates and their closest relatives. The tree was constructed using the weighted neighbor-joining tree building algorithm. A bootstrap analysis was performed using 100 resamplings and *Nocardia africana* as an outgroup. The bar represents a distance of 0.01 substitutions per nucleotide. The GenBank accession numbers of 16S ARN gene sequences are supplied into brackets.

Sequencing of the 16S rRNA genes and alignment with sequences retrieved from GenBank databases indicated that endophytic cacao isolates all belonged to the *Streptomyces* genus, except for Ref17, an isolate characterized by its long chains of ovoid spores, which was assigned to the genus *Actinomadura*. A phylogenetic tree was constructed based on 16S rRNA gene sequences for the 11 selected isolates and related species (Fig. 2). The phylogenetic dendrogram indicated a division of the endophytic bacteria into three distinct clades. Ref17 appeared to be closely related to *Actinomadura nitritigenes* because it shared a high 16S rRNA gene similarity of 99.9% with *A. nitritigenes* NBRC 15918^T^ (1 nt difference at 1,473 locations, Table S3). *Streptomyces* isolates were grouped into two distinct clades: eight isolates (Rec3, Ref8, Ref12, Ref16a, Ref16b, Ref16c, Ref22, and RefXX) were found to belong to the *S. violaceusniger* clade and were closely related to *S. geldanamycininus*, *S. rutgersensis*, *S. yatensis*, and *S. asiaticus*. The other clade included the isolates Rec1 and Ref20 (Fig. 2). Rec1 and Ref20 shared 98.9% similarity for the 16S rRNA gene (17 nt differences at 1,497 locations) and showed relatively low similarities with their closest phylogenetic neighbour (Table S3), which suggests that they represent undescribed species.

### Antagonistic activity of actinobacterial cacao endophytes

An isolate of each morphotype was tested for its capacity to inhibit the growth of plant pathogenic oomycetes, fungi, and bacteria. All endophytic isolates inhibited the growth of oomycetes and fungi. All of the endophytic bacteria isolates tested, except for the isolates Rec1, Ref17, and Ref20, also showed antibacterial activity against the bacterial plant pathogens *S. scabiei* and *A. tumefaciens* (Table 3). The isolates Rec3 and Ref8 were found to produce the wide spectrum antibiotic geldanamycin (44.3 μg mL^−1^ and 0.6 μg mL^−1^, respectively). However, none of the isolates showed an ability to impede the growth of the other actinobacterial cacao endophytes.

### Characterization of actinobacterial endophytes for additional traits linked to plant growth-promoting bacteria

The actinobacterial isolates exhibited several properties often associated with plant growth-promoting bacteria. All selected isolates, except for Ref17, were able to produce siderophores, as evidenced by an orange halo around bacterial colonies due to the removal of iron from Fe-CAS dye. Clear zones around colonies grown on PDYA were detected for all isolates as an indication of calcium phosphate solubilization. The 11 isolates were able to produce ACC deaminase (Table 3). The activity of ACC deaminase measured in the isolates ranged between 8.5 to 82.3 nmol α-ketobutyrate g bact. FW^−1^ h^−1^. All isolates, except for Rec1 and Ref20, produced IAA (Table 3).

Among the 11 selected endophytes, eight significantly promoted radish root growth. The most important growth-promoting activity was observed by inoculating radish seedlings with Ref8, which increased root dry weight by 53%.

## Discussion

Actinobacteria are common inhabitants of the plant rhizosphere and plant surfaces including the cacao rhizosphere (4) and cacao pods (37). However, although the presence of these bacteria inside cacao fruit has been poorly explored, several studies have revealed that these microorganisms live as endophytes in various plants (17, 18). Therefore, the present study focused on the actinobacterial population of both the cacao endocarp and seeds in order to compare populations within both fruit locations and identify actinobacteria that are disseminated to progeny. Forty-nine endophytic actinobacteria were successfully isolated. On the basis of morphological traits, 11 isolates were selected for further characterization. However, these isolates may not represent the actual diversity of actinobacterial endophytes present in the sampled cacao fruits because different species may have been gathered under the same morphotype and the isolation methods used here failed for certain endophytic actinobacteria. Diversity was higher within the seed actinobacterial community, and seed isolates included nine morphotypes that differed from endocarp isolates. Consistent with previous studies (11, 64), our results confirmed that endophytic communities vary within plant structures. Furthermore, as previously reported (44), no strict correlation was observed between the physiological and biochemical properties of plant-associated actinobacteria and their genetic relatedness.

Actinobacterial seed populations included isolates of *Streptomyces* and *Actinomadura*. Few studies have focused on the endophytic actinobacterial populations of fruits and seeds, although Coombs and Franco (13) demonstrated that actinobacteria colonize plants at the early stages of plant development and may be found in embryo and endocarp tissues. While *Streptomyces* species have been identified as the main endophytic actinobacteria for several plant species (21, 48), few studies have reported the isolation of endophytes belonging to the genus *Actinomadura*. However, *Actinomadura* has been isolated from another tree, the Australian native pine (29). In the present study, Ref17 isolates appeared to belong to the species *A. nitritigenes*, a nitrite-producing species (35). To the best of our knowledge, this is the first time that such a species has been described as an endophytic plant colonizer. A metagenomics analysis of the endophytic inhabitants of rice roots revealed that root endophytes may be involved in the entire nitrogen cycle including nitrification (54), and the same study suggested that bacteria exhibiting this trait are propagated to the offspring.

A high proportion of the morphotypes associated with seed isolates (six out of nine) lacked the ability to sporulate on all eight culture media tested (data not shown). In contrast to fungal communities, among which non-sporulating endophytic populations have been described (58), most of the previously characterized actinobacterial endophytes have exhibited sporulation abilities. Further studies are needed in order to determine whether cacao seed colonization promotes the loss of the sporulation phenotype or, alternatively, if the lack of sporulation ability is the result of genetic variations that translate into improved seed colonization ability.

Previous studies have shown that bacterial cacao endophytes belonging to the genus *Bacillus* promote the growth of the host (19, 41). Furthermore, *Bacillus* endophytes from vegetable crops may be efficiently used as the plant growth-promoting inoculants of cacao seedlings (40). In the present study, a reciprocal effect was shown because several actinobacterial cacao endophytes (eight out of the 11 isolates tested) were found to promote the development of radish roots. Independent of their ability to promote radish development, all cacao isolates showed properties often associated with plant growth-promoting rhizobacteria such as microbial antagonism, the production of siderophores and ACC deaminase, and phosphate solubilization ability. However, two out of the three isolates that did not significantly promote root development lacked the ability to produce the phytohormone IAA, whereas only one out of the nine IAA producers did not significantly promote radish development. The importance of microbial IAA in the promotion of root growth (45) is well-documented and differences in the plant growth-promoting effects of actinobacterial endophytes may be attributed to the inherent properties of the individual isolates (51).

In the present study, nearly 80% of the fruit cacao isolates belonged to the *S. violaceusniger* clade. Several members of this clade are known to produce geldanamycin (43), as were the isolates Rec3 and Ref8 described herein. Therefore, geldanamycin-producing bacteria appear to be distributed worldwide because they have been isolated from various countries (20) including Canada (63) and Cameroon (the present study). Several geldanamycin-producing *Streptomyces* strains contribute to plant health through their biocontrol and plant growth-promoting abilities (9, 16, 43). Geldanamycin-producing isolates also thrive as endophytes, thereby bringing a new perspective in their use as bacterial inoculants. All the isolates tested were resistant to geldanamycin. Furthermore, none of the endophytes tested had the ability to impede the growth of the other actinobacterial endophytes even if most of them inhibited the growth of another actinobacterium, *S. scabiei*. This result suggests that, as for other biological systems (38), antibiotic resistance is a colonization factor of cacao fruits. While several studies have focused on the antagonistic activity of endophytes against plant pathogens (7, 49), the effects of antibiosis in the establishment of plant endophytic communities have not yet been examined in detail. This question is of particular interest in the development of plant protection strategies.

## Supplementary Material



## Figures and Tables

**Table 1:** Morphological and cultural properties of 11 actinobacterial endophytes selected for characterization.

Sporulation	Isolate (no. of similar isolates)	Source	Morphological characteristics
Rectiflexible chains of spores	Rec1 (5)	Cacao pod	White substrate mycelium, yellow aerial mycelium, and white mass of spores on MS medium. Melanin production on ISP-7 medium.
	Ref20 (1)	Cacao seed	White substrate mycelium, white aerial mycelium, and white mass of spores on MS medium. Melanin production on ISP-7 medium.
Spiral chains of spores	Rec3 (29)	Cacao pod	Orange substrate mycelium, white aerial mycelium, and gray mass of spores on MS medium.
	Ref8 (1)	Cacao seed	Yellow substrate mycelium, white aerial mycelium, and gray mass of spores on MS medium. Melanin production on ISP-7 medium.
Long chains of ovoid spores	Ref17 (2)	Cacao seed	White substrate mycelium, white aerial mycelium, and white mass of spores on MS medium.
No sporulation	Ref12 (1)	Cacao seed	Yellow substrate mycelium and white aerial mycelium on MS medium.
	Ref22 (4)	Cacao seed	Orange substrate mycelium and white aerial mycelium on MS medium. Melanin production on ISP-7.
	Ref16a (3)	Cacao seed	Yellow substrate mycelium and orange aerial mycelium on MS medium.
	Ref16b (1)	Cacao seed	Yellow substrate mycelium and yellow aerial mycelium on MS and ISP-3 media.
	Ref16c (1)	Cacao seed	Yellow substrate mycelium and aerial mycelium on MS medium, yellow substrate mycelium and white aerial mycelium on IPS-3.
	RefXX (1)	Cacao seed	Yellow substrate mycelium and aerial mycelium on MS medium, white substrate mycelium and white aerial mycelium on IPS-3.

**Table 2:** Physiological traits associated with 11 actinobacterial isolates (+: growth, −: no growth, +/−: intermediary phenotype).

Characteristics	Ref8	Ref12	Ref16a	Ref16c	Rec3	Ref22	RefXX	Ref16b	Rec1	Ref20	Ref17
Substrate utilization											
Hypoxanthine	+	−	−	+	+	+	+	+	+	+	−
Pectin	+	−	+	+	+	+	−	−	+	+	+
Chitin	+	−	+	+	+	+	+	+	+	+	+
Chitosan	+	−	+	+	+	+	+	+	+	+	+
Growth condition											
NaCl (8%)	+	−	+	−	+	−	−	−	+	+	+
NaCl (10%)	+/−	−	−	−	+/−	−	−	−	+/−	−	+/−
CdCl_2_ (0.01%)	+/−	−	−	−	+	+	−	−	+/−	−	+
Thallium acetate (0.01%)	−	−	−	−	−	−	+/−	−	−	−	+
K_2_TeO_3_ (0.01%)	+/−	+/−	+	+/−	+/−	−	−	+	+	+	+
Crystal violet (0.0001%)	+/−	+/−	−	−	+/−	−	−	−	+	+/−	+
45ºC	−	−	−	−	−	−	−	−	+	−	−
Penicillin (10 IU mL^−1^)	+	+	+	+	+	+	+	−	+	+	+
Lincomycin (100 μg mL^−1^)	+/−	−	−	−	−	+/−	+	−	−	−	+
Streptomycin (100 μg mL^−1^)	−	−	−	−	−	+/−	+/−	−	−	−	−
Oleandomycin (1 μg mL^−1^)	+	−	+	−	+	+	+	−	+	+	−
Rifampicin (1 μg mL^−1^)	+	−	+	−	+/−	+	+/−	−	+/−	−	+
Vancomycin (50 μg mL^−1^)	−	−	+/−	−	−	+/−	+/−	−	−	+	+

**Table 3:** Characterization of endophytic actinobacterial isolates for traits commonly associated with plant growth-promoting bacteria (+: positive response, −: negative response).

Trait	Ref8	Ref12	Ref16a	Ref16c	Rec3	Ref22	RefXX	Ref16b	Rec1	Ref20	Ref17
Growth inhibition											
*P. megakaria*	+	+	+	+	+	+	+	+	+	+	+
*P. erythroseptica*	+	+	+	+	+	+	+	+	+	+	+
*F. oxysporum*	+	+	+	+	+	+	+	+	+	+	+
*B. cinerea*	+	+	+	+	+	+	+	+	+	+	+
*A. tumefaciens*	+	+	+	+	+	+	+	+	−	−	−
*S. scabiei*	+	+	+	+	+	+	+	+	−	−	−
P solubilization	+	+	+	+	+	+	+	+	+	+	+
Siderophore production	+	−	+	+	+	+	+	+	+	+	+
ACC deaminase act.* (±SD, *n*=2)	31.5±0.8	8.5±2.3	40.8±5.4	20.0±1.5	82.3±1.5	58.1±1.2	35.0±26.5	14.6±0.8	56.2±5.4	28.8±1.9	32.3±3.1
IAA biosynthesis	+	+	+	+	+	+	+	+	−	−	+
Root weight promotion (%±SD, *n*=3)	53.0±18.9	34.7±21.0	43.7±19.9	38.3±21.0	40.6±9.5	45.6±20.2	41.1±16.4	27.4±21.7	6.0±10.6†	24.8±23.7†	39.8±19.8

* ACC deaminase activity in nmol α-ketobutyrate g bact. FW^−1^ h^−1^.

† Value does not significantly differ from the non-inoculated control.
