# Supplementary material for: Morphological, Physiological, and Taxonomic Characterization of Actinobacterial Isolates Living as Endophytes of Cacao Pods and Cacao Seeds
Source: Microbes Environ. 2016 Mar 5;31(1):56–62. doi: 10.1264/jsme2.ME15146 (PMC4791117; doi:10.1264/jsme2.ME15146)
Supplement: Supplementary file 1 [file 31_56_s1.pdf]

Supplemental Table S1. Physiological tests for which no differences were observed between the isolated actinobacterial endophytes

Trait analyzed	Score
Substrate degradation	
Aesculin	+
Arbutin	+
Gelatin	+
Casein	+
Tween 80	+
Starch	+
Cellulose	+
Carbon source utilization	
L-arabinose	+
Cellobiose	+
Dextrin	+
Lactose	+
myo-Inositol	+
Raffinose	+
Xylose	+
Sodium propionate	+
L-leucine	+
L-tyrosine	+
L-methionine	+
L-serine	+
L-phenylalanine	+
L-valine	+
Growth under stress conditions	
NaCl (4%)	+
Phenol (0.1%)	+
pH 4.5	+
pH 9.0	+
4°C	–
25°C	+
37°C	+
Antibiotic resistance	
Cephaloridine (100 μg mL^{-1})	+
Geldanamycin (50 μg mL^{-1})	+
Kanamycin (50 μg mL^{-1})	–

Table S2. Physiological similarity index (%) between different isolates of endophytic actinobacteria

Isolate	Ref8	Ref12	Ref16a	Ref16c	Rec3	Ref22	RefXX	Ref16b	Rec1	Ref20	Ref17
Ref8	100										
Ref12	74	100									
Ref16a	84	79	100								
Ref16c	82	87	87	100							
Rec3	97	76	87	84	100						
Ref22	87	68	84	82	79	100					
RefXX	84	71	82	79	74	92	100				
Ref16b	76	87	82	95	79	76	79	100			
Rec1	92	74	84	82	97	79	71	76	100		
Ref20	89	82	92	92	89	82	74	84	89	100	
Ref17	87	74	82	76	89	79	76	71	84	84	100

Table S3. Closest phylogenetic neighbour with validly published name of the 11 selected isolates, based on the partial sequencing of 16 rRNA gene

Isolate	Closest neighbour	Percentage similarity (nt differences per number of locations)
Rec1	*Streptomyces heilongjiangensis* NEAU-W2^T	98.3% (25/1497)
Rec3	*Streptomyces hygroscopicus* subsp. *geldanus* NBRC 14620^T	99.9% (2/1427)
Ref8	*Streptomyces violaceusniger* Tu 4113	99.8% (3/1437)
Ref12	*S. violaceusniger* Tu 4113	99.8% (3/1436)
Ref16a	*S. violaceusniger* Tu 4113	99.6% (6/1503)
Ref16b	*S. violaceusniger* Tu 4113	99.3% (10/1494)
Ref16c	*S. violaceusniger* Tu 4113	99.8% (3/1489)
Ref17	*Actinomadura nitritigenes* NBRC 15918^T	99.9% (1/1473)
Ref20	*S. heilongjiangensis* NEAU-W2^T	98.0% (30/1504)
Ref22	*S. violaceusniger* Tu 4113	99.8% (3/1491)
RefXX	*S. hygroscopicus* subsp. *geldanus* NBRC 14620^T	99.7% (4/1437)

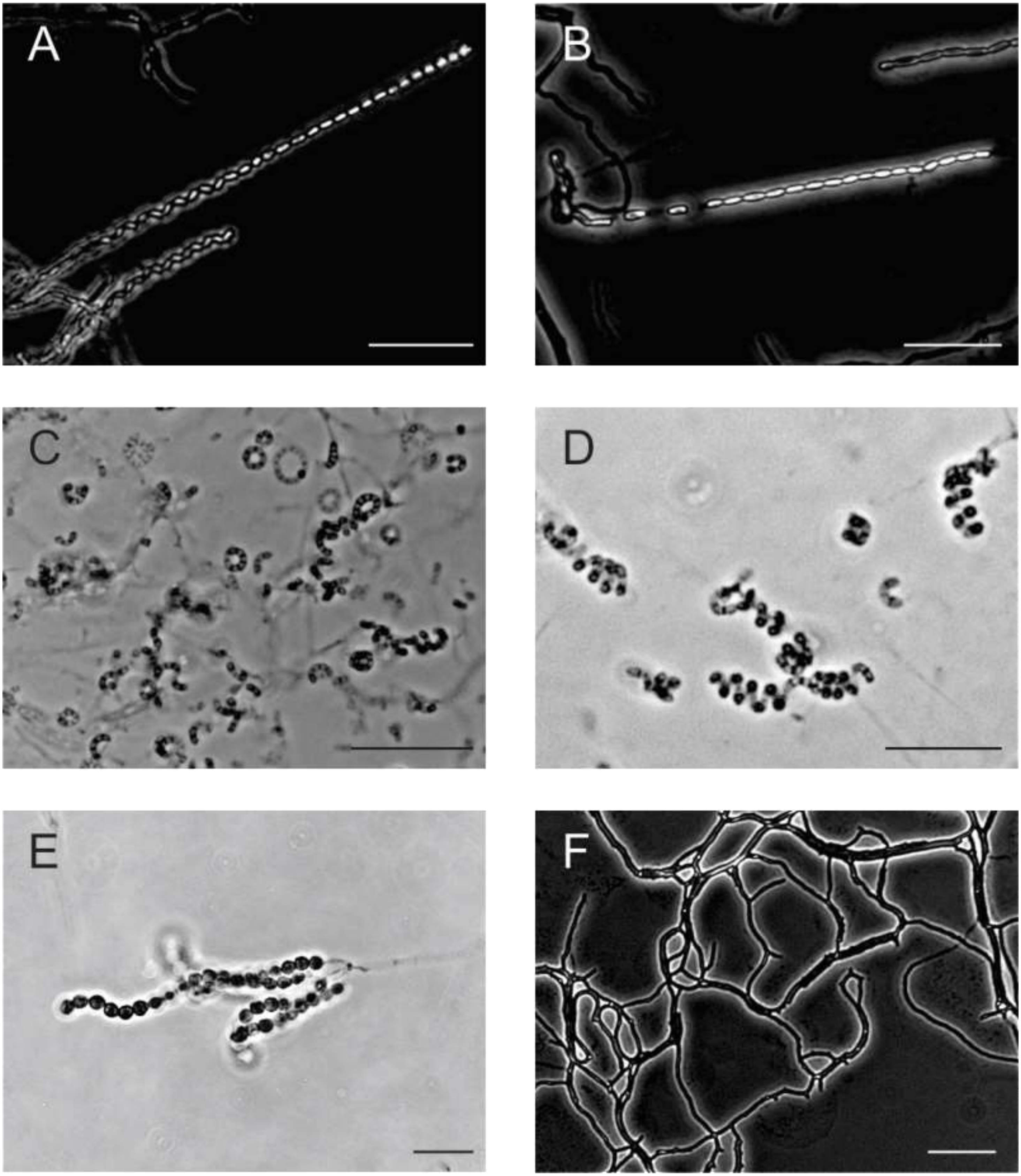

Supplemental Figure S1. Morphological characteristics of the 11 isolates observed with phase contrast microscopy after growth on ISP-2 medium. Isolate Rec1 (A) formed rectiflexible chains of spores occasionally exhibiting basal zigzag shape; isolate Ref20 (B) formed rectiflexible chains of spores; isolate Rec3 (C) and Ref8 (D) formed spiral chains of spores; isolate Ref17 (E) formed umbels carrying long chains of ovoid spores; while other isolates, such as Ref16c (F), did not sporulate. The bars represent 10 μm.
